# Pulse wave transit time technique for perioperative non-invasive hemodynamic monitoring

**DOI:** 10.1186/cc13329

**Published:** 2014-03-17

**Authors:** J Hruda, M Chobola, M Lukes, P Suk, J Klimes, V Sramek

**Affiliations:** 1 St Anna University Hospital and International Clinical Research Center, Brno, Czech Republic

## Introduction

The aim of this work is to evaluate perioperative hemodynamic monitoring for major abdominal surgery using the estimated continuous cardiac output (esCCO) technique available with the NIHON KOHDEN Vismo monitor as compared with the LiDCOrapid hemodynamic monitoring system.

## Methods

The esCCO technique is a novel noncalibrated non-invasive method of cardiac output monitoring. It is based on the estimation of pulse pressure from the speed of pulse wave propagation, which is measured as a time delay between the ECG R-wave and the corresponding peripheral pulse wave detected by pulse oximetry - the delay being called the pulse wave transit time (PWTT) [1]. LiDCOrapid estimates noncalibrated cardiac output (nCO) by pulse wave contour analysis from an arterial line and is a well-established monitoring modality in our department. The patients planned for elective major abdominal surgery with ASA score of III and expected operation duration over 90 minutes are routinely monitored by LiDCOrapid with a perioperative hemodynamic optimalization protocol. In accordance with the protocol, an arterial line was inserted and LiDCOrapid monitoring was started before the induction of anesthesia. At the same time, non-invasive esCCO monitoring was started.

## Results

Ten patients were monitored with a total of 141 esCCO-nCO measurement pairs. The correlation coefficient between esCCO and nCO was 0.65 and in the Bland-Altman difference analysis bias was + 1.2 l/minute and 95% limits of agreement were ± 2.6 l/minute. The change of cardiac output between two consecutive measurements detected by LiDCOrapid was detected by esCCO in 80% of cases.

## Conclusion

Hemodynamic monitoring with esCCO yields cardiac output values different from those measured by LiDCOrapid. The reliability of PWTT is questionable when systemic vascular resistance changes (as with any other noncalibrated cardiac output monitor), but it can still trace the trends of CO changes. Due to its non-invasiveness, esCCO might be the monitoring of choice for high-risk patients undergoing low-risk surgery.
